# Transient synchrony in delayed coupled neuronal networks

**DOI:** 10.1186/1471-2202-16-S1-P269

**Published:** 2015-12-18

**Authors:** Zahra G Esfahani, Alireza Valizadeh

**Affiliations:** 1 Department of Physics, Institute for Advanced Studies in Basic Sciences, Zanjan, Iran; 2 School of Cognitive Sciences, IPM, Niavaran, Tehran, Iran

## 

In this study, we propose that in a pool of neurons recurrently coupled through delayed synaptic connections transient patterns of synchrony can be observed due to the changing incoming stimuli, in continuance of some recent works [1]. Transient synchrony between spiking activity of the neurons has been reported in different sensory tasks e.g. visual and olfactory system [2,3].

We have shown that the critical role of the delay is to prepare connections that their synchronizing/desynchronizing effect changes when they receive different levels of stimuli [4,5]. In a suitable range of parameters, need not to be fine-tuned, an initially incoherent firing of the neurons can turn to coherent network oscillation when the mean input is changed -not necessarily increased--through sensory or control input (Figure 1). It is important to note that such an ability of the network to select frequencies of the oscillation is based on the presence of the delay in communication between neurons. In a network in which the components communicate instantaneously--with delays ignored--the neurons either spike synchronously or asynchronously depending on the connections properties and regardless of the value of the input current and the frequency of the spiking of the neurons.

**Figure 1:**
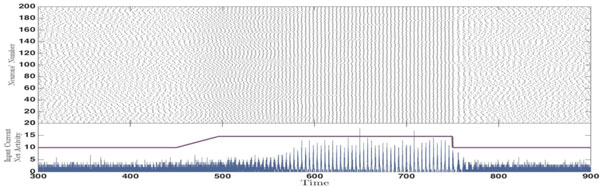
**Raster Plot of an all-to-all network of 200 homogenously coupled neurons with time dependent stimuli (the violet curve), which switches between asynchronous incoherent state to a synchronous state when the input is changed**. All the neurons are excitatory and external input to each neuron comprises a constant current chosen from a narrow normal distribution and an independent Gaussian white noise. The blue diagram presents the network activity.

## Conclusion

We have shown that the ability of a neural network to switch between coherent and incoherent firing, may be dependent on the delay in communication between neurons. It has been shown that two reciprocally coupled neurons can fire inphase if the delays lie in the region where the phase response curve of the neurons have negative slope, otherwise their firing is antiphase. In the larger networks where the neurons connect to several other neurons, inphase firing state remains stable where instead of antiphase state, several stable states appear. This is related to geometric *frustration *in condensed matter physics where a plenitude of distinct ground states are ensued by the lattice structure as in Ising system.
